# Supplementary material for: Power production and area usage of offshore wind and the relationship with available energy in the atmosphere
Source: PLoS One. 2025 May 2;20(5):e0321528. doi: 10.1371/journal.pone.0321528 (PMC12048160; doi:10.1371/journal.pone.0321528)
Supplement: S1 Table — Total installed capacity (Cap), area, average capacity factors, and averaging period in the dataset analyzed in this paper. [file pone.0321528.s001.pdf]

Table 1. Wind farms in the North Sea

Windfarm	Cap (MW)	Area (km^3)	CF (%)	Period
HornseaTwo	1386.0	462.0	41.4	2023 - 2024
HornseaOne	1218.0	407.3	46.1	2021 - 2023
Seagreen	1075.0	332.0	15.6	2023 - 2024
MorayEast	950.0	295.6	25.3	2022 - 2024
TritonKnoll	857.0	149.0	40.6	2023 - 2024
EastAngliaOne	714.0	162.8	46.9	2021, 2023
WalneyExtension	659.0	149.1	44.3	2020 - 2023
LondonArray	630.0	106.9	41.9	2020 - 2023
Beatrice	588.0	131.3	38.8	2020 - 2023
GwyntyMor	576.0	68.0	35.2	2020, 2022 - 2023
RaceBank	573.0	62.4	43.8	2020 - 2023
GreaterGabbard	504.0	146.1	39.1	2020 - 2023
Dudgeon	402.0	55.1	45.7	2020 - 2023
Rampion	400.0	56.3	41.1	2021 - 2023
WestOfDuddonSands	389.0	67.0	43.8	2020 - 2023
Galloper	353.0	113.7	47.3	2021 - 2023
SheringhamShoals	316.8	35.0	36.5	2022 - 2023
Lincs	270.0	38.9	41.7	2020 - 2023
BurboBankExtension	254.0	39.6	40.1	2020 - 2023
HumberGateway	219.0	27.0	42.6	2020 - 2023
WestermostRough	210.0	34.9	46.0	2020 - 2023
Walney1	184.0	27.1	36.8	2020 - 2023
Walney2	184.0	45.9	43.8	2020 - 2023
RobinRigg	174.0	18.3	36.0	2020 - 2023
GunfleetSands	173.0	15.8	35.4	2020 - 2023
Ormonde	150.0	9.9	39.8	2020
Aberdeen	96.8	20.0	36.8	2020
BurboBank	90.0	10.0	31.8	2020 - 2023
Barrow	90.0	10.0	31.7	2021 - 2022
Kincardine	50.0	20.0	19.3	2020 - 2023
HywindScotland	30.0	15.4	50.8	2020 - 2023

Total installed capacity (Cap), area, average capacity factors, and averaging period in the dataset analyzed in this paper.
