# Supplementary material for: Power production and area usage of offshore wind and the relationship with available energy in the atmosphere
Source: PLoS One. 2025 May 2;20(5):e0321528. doi: 10.1371/journal.pone.0321528 (PMC12048160; doi:10.1371/journal.pone.0321528)
Supplement: S1 Appendix — A description of the method to identify wind farms that do not operate at full capacity. [file pone.0321528.s002.pdf]

Identifying wind farms not operating at full capacity

November 28, 2024

To investigate whether power production in wind farms is limited by atmospheric energy input, it is important that the wind farms included in the study are operating at full capacity and not limited by some technical issues or other reasons. The simple visual inspection that is done for each wind farm is illustrated in this appendix. The half-hourly timeseries of a wind farm operating at full capacity is shown in Figure 1, while the timeseries for the four wind farms excluded from the study are shown in Figures 2 - 5. In Figure 1, it is clear that the peak of the power production is close to the total capacity of the wind farm. Some periods of low power production typically occurs during summer. The Seagreen wind farm (Figure 2) is seen to almost never have a power production close to its capacity. Seagreen was commissioned in October 2023, and in Figure 2 the increase in power production up to the commissioning is clearly seen. But even after October 2023 the power production is very low compared to the full capacity. The Moray East wind farm (Figure 3) has long periods in which clearly large parts of the wind farm are shut down. In the spring of 2022 and in 2024, the production seams low compared to the total capacity. The Sheringham Shoals wind farm (4) also has periods in which parts of the wind farm are turned off, and the Kincardine wind farm (5) has clearly almost never operated at full capacity.

Seagreen, Moray East, Sheringham Shoals and Kincardine wind farms are excluded from the study because they seem to be limited by other reasons than the atmospheric energy input. Periods of low power production also occur in the other wind farms included in the study, but for these wind farms it was possible to identify a period of full operation that lasted for a year or more.

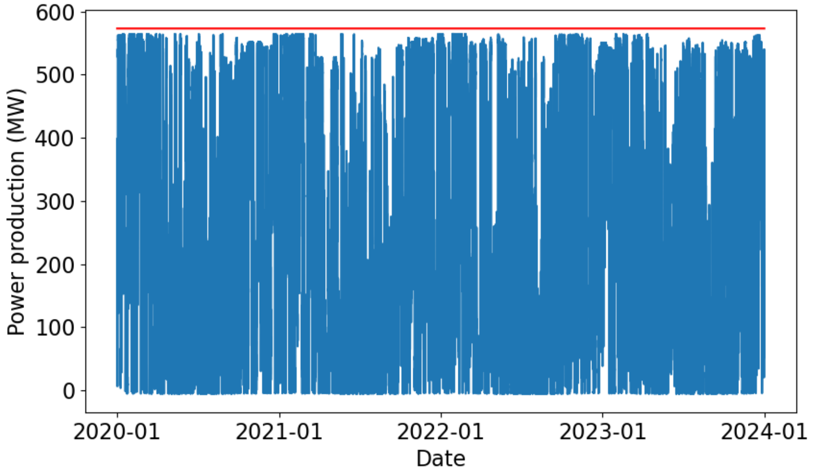

Figure 1: How hourly power production of Racebank wind farm in blue and total installed capacity in red

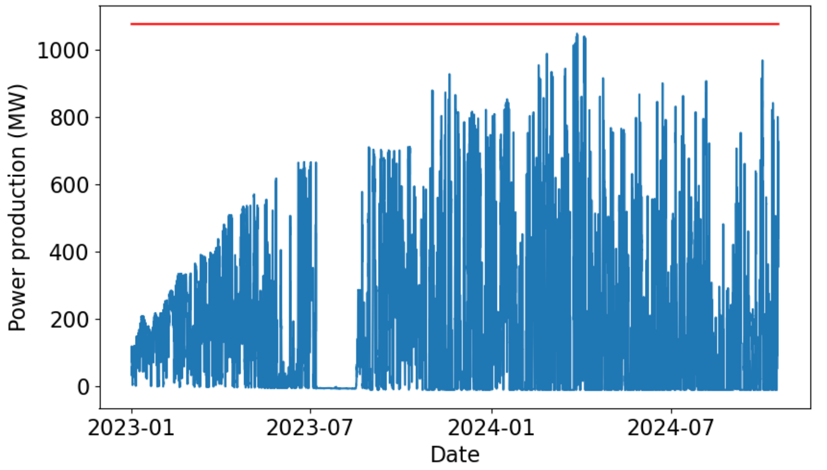

Figure 2: How hourly power production of Seagreen wind farm in blue and total installed capacity in red

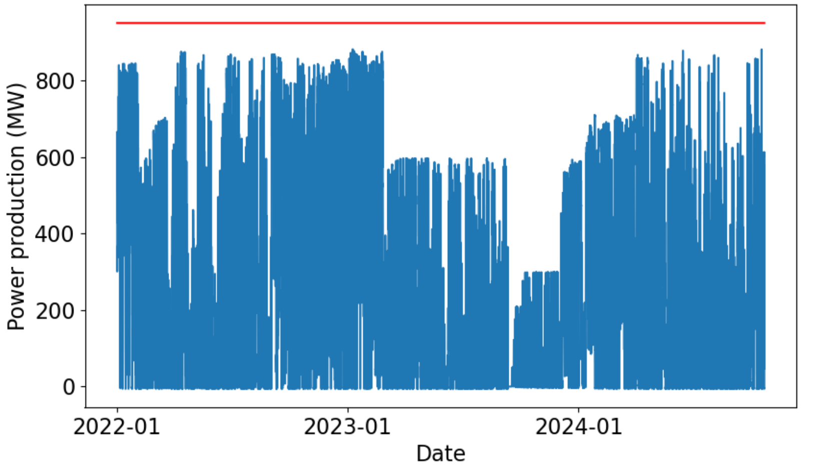

Figure 3: How hourly power production of Moray East wind farm in blue and total installed capacity in red

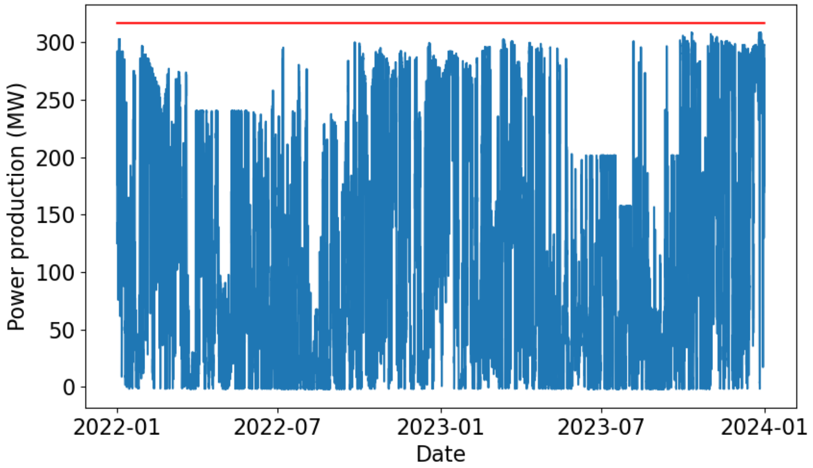

Figure 4: How hourly power production of Sheringham Shoals wind farm in blue and total installed capacity in red

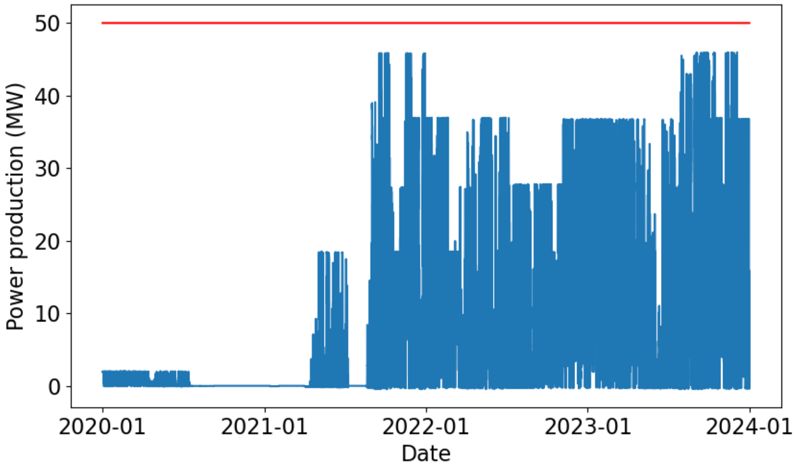

Figure 5: How hourly power production of Kincardine wind farm in blue and total installed capacity in red
