# Supplementary material for: Power production and area usage of offshore wind and the relationship with available energy in the atmosphere
Source: PLoS One. 2025 May 2;20(5):e0321528. doi: 10.1371/journal.pone.0321528 (PMC12048160; doi:10.1371/journal.pone.0321528)
Supplement: Attachment — Submitted filename: CommentToAuthors.pdf [file pone.0321528.s003.pdf]

The paper is well-written, and it seems of value for wind energy community both scientific and industry. Please see comments below:

Small edits:

1. Line 22 p1/14: within 2050 → by 2050

2. future area "needs" for offshore wind. → "needed" for offshore wind.

3. Please give a simple and short definitions to capacity density, capacity factor for readers outside of the field.

4. Table 1 caption mentions that the table has the coordinates and locations of the farms but there is no such information on the table. This table can also be moved to supplementary materials. You should also define cf202X meaning in the caption such as "Capacity Factor for 202X (cf202X)".

5. Please discuss the details of what is in figure 2 in caption. Figure 2 has parts a, and b. You should have a brief explanation for these in the caption.

6. In equation 9, what is ndl?

7. You don't explain what the mixing arrows are in the figure 4.

Please see comments below:

1. "However, since W depends on an average along the circumference of the wind farm and Z on an average over the wind farm area, it is unlikely that they will vary significantly with area." → does this mean that you are ignoring the role of atmospheric stability in your calculations?

2. Can you provide steps to how you rearranged 15 to 16?

3. "If the installed capacity is zero (no wind farm), Eq 15 still applies, but the input of energy will be balanced by dissipation only. When increasing the installed capacity, the power production will increase until it reaches a limit, given by the input of energy." → please clarify this by determining why these sentences are true. For instance, "when increasing the installed capacity, the power production increase, [add what would change in Eq 15], and therefore it reaches a limit.

4. Line 229, page 8: "assuming that all wind farms are shaped as squares", this is a big assumption as in reality the wind farms are hardly shaped as a perfect square. What uncertainties do you think this assumption adds to your calculations? Please add a few sentences discussing this.

5. In page 8, when you remove wind farms with smaller capacity (<200MW) you get better correlation. Please consider explaining a reason for this. And specify how many of the wind farms are getting removed by removing the small farms (what percent of the wind farms are removed)?

6. In Discussion, you mention "Wind farms with a total capacity below 200 MW (shown in gray in Fig 5 b and c) probably operate with a lower efficiency and therefore the assumption of constant WP and ZP is not valid for these wind farms." → How did you decide on threshold of 200MW as for farms that do not perform with high efficiency?

7. Table 2, are you using ZP = 0.3 divided by 0.8? If so, why is that? On page 9 you mentioned that it could vary between these two numbers as upper and lower limits.
