# Supplementary material for: Power production and area usage of offshore wind and the relationship with available energy in the atmosphere
Source: PLoS One. 2025 May 2;20(5):e0321528. doi: 10.1371/journal.pone.0321528 (PMC12048160; doi:10.1371/journal.pone.0321528)
Supplement: Attachment — Submitted filename: ResponsToReviewers.pdf [file pone.0321528.s005.pdf]

In this document copied text from editor or reviewers are presented in bold while my answers are in italics.

Edits on the revision requested

Thank you for uploading your study's underlying data set. Unfortunately, the repository you have noted in your Data Availability statement does not qualify as an acceptable data repository according to PLOS's standards.

At this time, please upload the minimal data set necessary to replicate your study's findings to a stable, public repository (such as figshare or Dryad) and provide us with the relevant URLs, DOIs, or accession numbers that may be used to access these data. For a list of recommended repositories and additional information on PLOS standards for data deposition, please see https://journals.plos.org/plosone/s/recommended-repositories.

I have solved this as follows:
The data needed to replicate the study is power production data, installed capacity and area for all wind farms and atmospheric data to derive the result shown in Figure 6.

The installed capacity, area and capacity factors for all wind farms are given in supporting material, S1_Table.

The capacity factors are derived from half-hourly power production data. Such data can be downloaded from Entsoe Transparency Platform and Elexon. The Elexon data can freely be redistributed to others. However, there are restrictions on the data from Entsoe Transparency Platform, and this data are not allowed to redistribute or use without permission from the primary data owners. I was not aware of this and simply assumed that data that was easily downloadable was also free to use. Unfortunately, this is not the case. I have not been able to get permissions from the data owners, and therefore I have been forced to remove the data downloaded from Entsoe Transparency Platform from the analysis. The analysis presented in the paper is now only based on data downloaded from Elexon. The dataset is therefore reduced from 38 to 31 wind farms.

The conclusions of the original manuscript still hold. The reduction in the amount of available data has led to a more detailed analysis of the data. An appendix have been added to the supporting material to explain the visual inspection of the timeseries of power production to find the wind farms that are not operating at full capacity. The methods for analysis of the data are the same as before, but now we remove data from wind farms that do not operate at full capacity. Uncertainties are now somewhat larger,

but the concluding values from the original manuscript are within the span of uncertainties in the revised manuscript.

The dataset necessary to replicate the findings of the study are uploaded to Dryad. The dataset will be kept private until this paper is accepted. The data can be accessed using this link: https://datadryad.org/stash/share/MkGAJBSvPKVA_YiYuw1p7-1y1diCuIzNToIv0fNF1sU

The final link to the power production dataset when released for public access is: https://doi.org/10.5061/dryad.w6m905qzk .

The atmospheric data needed to do the calculations of conversion from potential to kinetic energy within the atmospheric boundary layer and replicate the plot shown in Figure 6, can be downloaded from https://psl.noaa.gov/data/gridded/data.ncep.reanalysis2.html. More direct links to the exact files needed are given below. The data that is necessary to replicate the calculations in the manuscript are:

Air temperature on pressure levels: datafile = air.YYYY.nc
Geopotential height on pressure levels: datafile = hgt.YYYY.nc
Omega (vertical velocity in pressure coordinates on pressure levels:
datafile = omega.YYYY.nc
Eastward wind component at pressure levels: datafile = uwnd.YYYY.nc
Northward wind component at pressure levels: datafile = vwnd.YYYY.nc

In the calculations in the manuscript we have used the years 2020, 2021 and 2022. In the datafile name YYYY is replaced by the year.

The data on pressure levels can be downloaded from https://psl.noaa.gov/thredds/catalog/Datasets/ncep.reanalysis2/pressure/catalog.html

In addition to the data on pressure levels, the surface pressure is also needed.
Pressure at surface: datafile = pres.sfc.YYYY.nc

Surface pressure data can be downloaded from https://psl.noaa.gov/thredds/catalog/Datasets/ncep.reanalysis2/surface/catalog.html

……….

We note that your manuscript is not formatted using one of PLOS ONE's accepted file types. Please reattach your manuscript as one of the following file types: .doc, .docx, .rtf, or .tex (accompanied by a .pdf).

If your submission was prepared in LaTex, please submit your manuscript file in PDF format and attach your .tex file as “other.

Correct file types are now uploaded.

Journal requirements

1. Please ensure that your manuscript meets PLOS ONE's style requirements, including those for file naming. The PLOS ONE style templates can be found at

https://journals.plos.org/plosone/s/file?id=wjVg/PLOSOne_formatting_sample_main_body.pdf

and

https://journals.plos.org/plosone/s/file?id=ba62/PLOSOne_formatting_sample_title_authors_affiliations.pdf

I have used the PLOS ONE latex template to prepare the manuscript. I have checked against the pdf templates given, and I believe the manuscript meets the PLOS ONE style requirements.

2. Thank you for stating the following in the Acknowledgments Section of your manuscript:

“This work has been funded by the Norwegian Seafood Research Fund grant no. 901916.”

We note that you have provided funding information that is currently declared in your Funding Statement. However, funding information should not appear in the Acknowledgments section or other areas of your manuscript. We will only publish funding information present in the Funding Statement section of the online submission form.

Please remove any funding-related text from the manuscript and let us know how you would like to update your Funding Statement.

The funding related text is removed from the Acknowledgement section and replaced by a sentence where I thank the reviewers for their work, and acknowledgements of the data providers.

3. We note that Figures 1 and 6 in your submission contain [map/satellite] images which may be copyrighted. All PLOS content is published under the Creative Commons Attribution License (CC BY 4.0), which means that the manuscript, images, and Supporting Information files will be freely available online, and any third party is permitted to access, download, copy, distribute, and use these materials in any way, even commercially, with proper attribution. For these reasons, we cannot publish previously copyrighted maps or satellite images created using proprietary data, such as Google software (Google Maps, Street View, and Earth). For more information, see our copyright guidelines: http://journals.plos.org/plosone/s/licenses-and-copyright.

Both Figures1 and 6 were created by me, and they are not taken from any previously copyrighted maps or satellite images. I have used the Cartopy Python package to create the maps and plotted in wind farm locations in Fig 1 and conversion from potential to kinetic energy (estimated from the NCEP-DOI dataset) in Fig 6. The figures do not include any satellite images. Therefore, there are no problems with copyrights on these figures.

Reviewer's Responses to Questions

3. Have the authors made all data underlying the findings in their manuscript fully available?

The PLOS Data policy requires authors to make all data underlying the findings described in their manuscript fully available without restriction, with rare exception (please refer to the Data Availability Statement in the manuscript PDF file). The data should be provided as part of the manuscript or its supporting information, or deposited to a public repository. For example, in addition to summary statistics, the data points behind means, medians and variance measures should be available. If there are restrictions on publicly sharing data—e.g. participant privacy or use of data from a third party—those must be specified.

Reviewer #1: No

Reviewer #2: Yes

All power production data necessary to replicate the findings in the study are now uploaded to a public repository. It is kept in private mode until acceptance of this manuscript, but can be downloaded from https://datadryad.org/stash/share/MkGAJBSvPKVA_YiYuw1p7-1y1diCuIzNToIv0fNF1sU

Atmospheric data needed to replicate the plot in Figure 6 can be downloaded from https://psl.noaa.gov/data/gridded/data.ncep.reanalysis2.html. See detailed description of the downloading process in the Data Availability Statement, and above in this document.

Response to reviewers:

Thanks to the reviewers for some very good points and questions. This made me think carefully through the effect of the assumptions made and the uncertainties they bring into the theory. I believe especially the discussion and conclusion is significantly improved based on the input from the reviewers.

Reviewer #1

The authors have done an excellent job; however, here are my comments:

1) In Table 1, in the Dudgeon row under the Area column, there is an extra "N".

The N is removed. Following the suggestion from reviewer #2, the table is moved to supporting material.

2) Please clarify which unit of measurement "WM^-2" refers to in Figure 2.

This is a typo. It should be Watts per square meter (Wm^{-2}). This is fixed.
In addition, the figure 2 has changed as I discovered that the y-axis labels were wrong. The power density is in the upper panel (a) and the capacity density is in the lower panel (b). This is now correct.

3) The conclusions repeat the same idea regarding the C/A ratio; please improve the conclusions.

The conclusion is changed. The idea behind the C/A ratio is toned down, but not completely removed as it is an important conclusion of the paper. The conclusion now focuses more on the result that power production of the largest wind farms is limited by the atmospheric energy input. It also lists power densities that can be expected from future wind farms. Finally, it points out that estimates of power potential must include an atmospheric energy budget, because this is the limiting factor for power production.

4) I believe the paper should include a discussion section. Since this is a methodological proposal, it should have an extensive discussion. Please add this section.

I interpret the reviewer's comment as he/she is not satisfied with the discussion in the paper. The discussion section in the revised manuscript is improved by including a thorough discussion of the major assumptions that the theory is based on and of the uncertainties in the estimates of power density. These are important topics to be discussed for a methodical paper as pointed out by the reviewer. The improvement of the discussion section is done also on the basis of the comments from reviewer #2.

Reviewer #2

The paper is well-written, and it seems of value for wind energy community both scientific and industry. Please see comments below:

Small edits:

1. Line 22 p1/14: within 2050 → by 2050

The text now reads "by 2050"

2. future area "needs" for offshore wind. → "needed" for offshore wind.

The text now reads "needed" for offshore wind

3. Please give a simple and short definitions to capacity density, capacity factor for readers outside of the field.

The second paragraph in the introduction of the revised manuscript now gives a short definition of capacity density, power density and capacity factor.

4. Table 1 caption mentions that the table has the coordinates and locations of the farms but there is no such information on the table. This table can also be moved to supplementary materials. You should also define cf202X meaning in the caption such as "Capacity Factor for 202X (cf202X)".

The table is now moved to supplementary material S1_table. The mentions of coordinates and locations are removed. Since the analysis in the revised manuscript is based only on the Elexon data, some more data are downloaded. Some of the newest large wind farms have been fully operational after 2022, and therefore data up to October 2024 is downloaded. The data is now covering somewhat different time periods, and therefore only the mean capacity factor for each wind farm is presented in the table. The notation cf202X does therefore not exist any more.

5. Please discuss the details of what is in figure 2 in caption. Figure 2 has parts a, and b. You should have a brief explanation for these in the caption.

The upper and lower panel in the figure is now marked a) and b), and the caption simply states that "a) Power density and b) capacity density for the wind farms presented in Fig 1 and S1 Table, plotted against wind farm area." This should be ok given that power density and capacity density are defined in the introduction (see comment 3 reviewer #2). I must also inform that in Figure 2, power density and capacity density had switched places in the original manuscript. This is now fixed.

6. In equation 9, what is ndl?

n is the outward unit normal vector to the circumference of the wind farm and dl is an incremental distance along the circumference. ndl (n is marked as a vector in the manuscript)

is the product of the normal vector n and the distance dl. I have now added an explanation of this in the manuscript just after equation 9. In addition, I have also added an explanation of how the line integral (second term on lhs of equation 9) is obtained using Green's theorem.

7. You don't explain what the mixing arrows are in the figure 4.
I have now added an explanation to the mixing arrows in the caption to figure 4.
Please see comments below:
1. "However, since W depends on an average along the circumference of the wind farm and Z on an average over the wind farm area, it is unlikely that they will vary significantly with area." → does this mean that you are ignoring the role of atmospheric stability in your calculations?
No, the role of atmospheric stability is not ignored. The effect of atmospheric stability is embedded in the parameters W_P and Z_P.

The atmospheric stability affects the turbulent transfer of kinetic energy within the ABL. This is how it works in the theory that is developed in the paper:

The available energy is defined as the energy contained within the full height of the ABL. The source of the energy in the ABL is the incoming horizontal winds (WC/A) and the work done by pressure forces (Z). The vertical turbulent transfer of kinetic energy is set to zero on top of the ABL, because turbulence is typically negligible above the boundary layer. Therefore, it can be no turbulent flux of kinetic energy into the ABL from above, W and Z describe the energy balance in the ABL by the linear function of C/A (Eq 12).

However, for wind farm power production, turbulent exchange is important as turbulence transfer kinetic energy from the upper parts of the ABL into the wind farm below. The turbulence does not contribute to the input of kinetic energy to the ABL as a whole, but turbulent transfer of kinetic energy within the ABL is important for the power production in wind farms. W_P and Z_P determine the power production through equation 17. Since turbulent entrainment of kinetic energy is important, and W_P and Z_P are determined by fitting to power production data, the effect of atmospheric stability is embedded in these parameters. A section discussing this is now included in the discussion.

2. Can you provide steps to how you rearranged 15 to 16?
Getting from equation 15 to 16 is done as follows.
First equation 15

$$W\frac{C}{A} + Z = \frac{D + P}{A}$$

This is equivalent to

$$W\frac{C}{A} + Z = \frac{P}{A}(\frac{D}{P} + 1)$$

which leads to equation 16

$$\frac{P}{A} = \frac{1}{1+\frac{D}{P}}(W\frac{C}{A} + Z)$$

I have chosen not to include this extra step in the manuscript

3. "If the installed capacity is zero (no wind farm), Eq 15 still applies, but the input of energy will be balanced by dissipation only. When increasing the installed capacity, the power production will increase until it reaches a limit, given by the input of energy." → please clarify this by determining why these sentences are true. For instance, "when increasing the installed capacity, the power production increase, [add what would change in Eq 15], and therefore it reaches a limit.

This is a good question that is very central to the conclusion of the paper. I realize that the discussion of this was too simplified in the original manuscript. In the revised manuscript a detailed explanation of the variability of D/P is included in connection with Equation 16, and this topic is also discussed in the first paragraph of the discussion.

The basic mechanism is as follows:
If P=0, the input of energy is balanced by D only. Then D=D0, and D/P is infinite. If you install one turbine within an area of for instance 100 km2, then the power production is dP and D=D0+dD. If we consider a large area, then dD<<D0 and D/P=D0/dP. If we add one more turbine, D/P=D0/2dP. So, if the power production is much smaller than what is available for power production, then D/P will decrease with an increase in the installed capacity. Therefore, one cannot assume that D/P is constant when the installed capacity is small. However, when the installed capacity and the corresponding power production is large enough to be limited by the available energy, the situation is different. In this case, an increase in the installed capacity will not lead to an increase in power production, because the energy is not available. Then the assumption that D/P is constant is more realistic.

Please read the paragraph right after equation 16, which explains the changes in equation 16 when power production is increased from zero up to a level where it is limited by the atmospheric energy input.

4. Line 229, page 8: "assuming that all wind farms are shaped as squares", this is a big assumption as in reality the wind farms are hardly shaped as a perfect square. What uncertainties do you think this assumption adds to your calculations? Please add a few sentences discussing this.

I have added a discussion about this in the second paragraph of the discussion section. The basic assumption is not which shape that is chosen, but that all wind farms are assumed to have the same shape. For basically all shapes, C/A will be proportional to $1/\sqrt{A}$. For a square, $\frac{C}{A} = 4/\sqrt{A}$, and for a circle $\frac{C}{A} = 2/\sqrt{\pi A}$. The values of WP and ZP are found by fitting a function of the form $W_P X + Z_P$ to the data, where X represent C/A for the chosen shape. However, since X is proportional to $1/\sqrt{A}$ for any shape, the choice of shape does not affect the correlation to the

data. The shape only affects the value of WP, since C/A varies with shape for a constant area. The choice of shape does not affect P/A or ZP. What causes uncertainty is that all wind farms cannot be represented with the same shape. Variability in shape leads to deviations in how C/A relates to $1/\sqrt{A}$, and this leads to deviations to the curve estimated by assuming all wind farms are of similar shape. An estimate of the error is given in the Discussion section. For wind farms less than 50 km2, the error can be larger than 0.4 Wm-2, while for wind farms around 500 km2, the error is in the order of 0.1 Wm-2. The error will tend to zero for an increasing area. The error due to variations in shape is therefore equal to or smaller than the error estimated from equation 19. Please read the second paragraph in the discussion section for more details on this discussion of errors caused by the assumption on shape.

5. In page 8, when you remove wind farms with smaller capacity (<200MW) you get better correlation. Please consider explaining a reason for this. And specify how many of the wind farms are getting removed by removing the small farms (what percent of the wind farms are removed)?

The reason for this is connected to the assumption that D/P is constant- The background for this assumption is explained above (question number 3, reviewer #2). It is also explained in the paragraph following equation 16 and in the first paragraph in the discussion. The correlation between equation 16 and data assumes that D/P can be regarded as constant. However, for small wind farms that are not limited by the atmospheric energy input, assuming constant D/P is not realistic. This is why the correlation is better for the large wind farms. An alternative explanation is that errors are larger for small wind farms, as shown in the analysis of errors due to the assumption of wind farm shape.

In the caption for figure 5, I now also list how many of the wind farms are included in the correlation. There are a total of 31 wind farms. Four wind farms are excluded because the power production compared to the full capacity reveals that they are not operating at full capacity, Of the 27 wind farms that remain, 23 are larger than 100 MW and 18 are larger than 200 MW.

6. In Discussion, you mention “Wind farms with a total capacity below 200 MW (shown in gray in Fig 5 b and c) probably operate with a lower efficiency and therefore the assumption of constant WP and ZP is not valid for these wind farms.” → How did you decide on threshold of 200MW as for farms that do not perform with high efficiency?

The point is to show that the correlation between theory and data increases for increasing capacity. In Figure 5 the correlation for all wind farms and wind farms larger than 100 MW and 200 MW are shown. The correlation coefficient is 0.52 for all wind farms, while it increases to 0.84 for wind farms above 100MW. Wind farms above 200 MW have a correlation of 0.77. Thus, correlation is quite good for wind farms above 100MW. In the new dataset used in this revised manuscript (data from Entsoe Transparency Platform is removed, see above), the correlation is best for wind farms

above 100MW, but estimates of ZP are quite similar when using wind farms above 100MW and 200MW. Therefore, I have now chosen to use wind farms above 100MW, and the results for wind farms above 200MW are well inside the uncertainty span.

7. Table 2, are you using ZP = 0.3 divided by 0.8? If so, why is that? On page 9 you mentioned that it could vary between these two numbers as upper and lower limits.

In the original table I used the "/" to separate upper and lower limits. This looked like a division and could therefore lead to misunderstandings. Now I present the estimated values with uncertainties in parenthesis. Due to the removal of data from Entsoe Transparency Platform, the upper and lower values have changed, and ZP are now presented as 0.78 ± 0.58 W/m2. In the notation of the table this will be presented as 0.78 (0.58).
