# Supplementary material for: Power production and area usage of offshore wind and the relationship with available energy in the atmosphere
Source: PLoS One. 2025 May 2;20(5):e0321528. doi: 10.1371/journal.pone.0321528 (PMC12048160; doi:10.1371/journal.pone.0321528)
Supplement: Attachment — Submitted filename: ResponsToReviewers_auresp_2.pdf [file pone.0321528.s006.pdf]

Reviewer #1: The authors followed all the suggestions requested so, my recommendation is to accept the manuscript.

Thank you! I have worked hard to come up with solid responses to your comments and this has clearly improved the quality of the manuscript.

Reviewer #3: The paper should make some improvements:

1. The innovation is not clear in the beginning. The reason to publish it is that the added value of this work for the global S&T audience (introduction) needs more definition in the introduction.

The work presented in the manuscript derives a fundamental theory for the power production of offshore wind. The theory is based on the governing equations for the atmospheric momentum balance and arrives at a simple relation between power density and the area of a wind farm. It is well known that power density decreases with increasing area, but until now it has not been clear how large a wind farm must be for this process to be significant. From the developed theory and its agreement with actual measured power production, this is significant for existing wind farms ranging in size from almost zero to more than 400 km2.

In addition, from the result one can conclude that the limiting factor for power production is the input of energy from the atmosphere. There are many recent estimates of power potential that do not consider the atmospheric energy budget. From the results presented we now know that the atmospheric energy budget is vital. It is the limiting factor and therefore must be part of a realistic estimate of power potential.

Finally, a fundamental analytical theory that agrees with data is important. It brings understanding and insight into the problem.

I have added these sentences to the end of the introduction.

"Estimates on wake effects on power production are usually performed with complex numerical models [4-6]. Numerical models are highly important, but it can be difficult to extract physical understanding from complex numerical models. The analytical theory derived in this paper isolates first-order processes and provides understanding of the physics controlling the power production of offshore wind. The theory agrees with observations and the results of numerical models and, therefore, deepens the insight into the main physics of the problem."

2. The sustainable siting parameter beyond the wind potential should be included in the state-of the art (environment, regulations, other uses, etc). Several very recently published scientific works are available.

This is good advice, and I have added about one page in the introduction to cover these topics. I have focused on the environmental effects caused by wind wakes. This is natural since the energy budget analysis in the manuscript is essentially a method to quantify the effect of wakes. Wakes have effects on the ocean circulation and ecosystem and may also cause conflicts between wind farm operators and countries. This is now described in the introduction and several recent publications are included.
